# The Application of Emodin Treatment on Nasopharyngeal Carcinoma Therapy

**DOI:** 10.3390/biomedicines12030486

**Published:** 2024-02-21

**Authors:** Chung-Chun Wu, Mei-Shu Chen, Jen-Yang Chen

**Affiliations:** 1 Translational Cell Therapy Center, Department of Medical Research, China Medical University Hospital, Taichung City 404447, Taiwan; 2 National Institute of Cancer Research, National Health Research Institutes, Zhunan 350401, Taiwan; meishu@nhri.edu.tw

**Keywords:** nasopharyngeal carcinoma, natural compound, EBV, NPC therapy, emodin

## Abstract

Nasopharyngeal carcinoma (NPC) is a malignancy prevailing in Taiwan, Hong Kong, Southern China, Southeast Asia, and North Africa. Although early-stage NPC responds well to the primary treatment of radio-chemotherapy, the mortality rate of advanced NPC remains high. Therefore, developing new therapies for nasopharyngeal carcinoma is an urgent task. Emodin is an anthraquinone derivative mainly found in *Rheum palmatum*. Emodin has been found to possess many anti-cancer functions against various types of cancers, but they are less discussed in the treatment of NPC. This review organized the different studies about the anti-NPC activity of emodin and discussed the potential and challenges of emodin treatment in NPC therapy.

## 1. Nasopharyngeal Carcinoma (NPC)

### 1.1. Introduction of NPC

Nasopharyngeal carcinoma (NPC) commonly occurs in the epithelial cells of the nasopharynx, which is the uppermost part of the pharynx, located posterior to the nasal cavity. NPC commonly appears in adults in their 40s and 50s. Males are more frequently diagnosed than females. NPC has distinctive features in its epidemiology, etiology, and tumor biology. Regarding geographic distribution, epidemiological surveys revealed that NPC is an uncommon cancer with an incidence rate of 1 in 100,000 yearly worldwide [1]. However, the incidence rates of this malignancy are relatively higher in endemic regions, including Taiwan, Hong Kong, Southern China, Southeast Asia, and North Africa, which is 10–30 per 100,000 yearly [2,3]; it is less than 1 in 100,000 per year in Western countries and other low incidence regions [4,5,6]. For the contributions of gender and age, the incidence of NPC is notably higher in men than women, with a gender ratio of 3:1. The incidence rate escalates with increasing age at diagnosis, ranging from less than 0.1 in younger age groups to 0.9 in older populations per 100,000 individuals per year [7].

### 1.2. The Etiology of NPC

Since the first case of nasopharyngeal carcinoma was published in 1901 [8], the causes of its occurrence are still not very clear. To date, after extensive studies, three factors, genetics, diet, and viral factors, are considered in the primary etiology of nasopharyngeal carcinoma. In addition to geographic distribution, genetic factors play a significant role in the occurrence of nasopharyngeal carcinoma. In Southern China, studies indicate that more than 5% of patients diagnosed with nasopharyngeal carcinoma have a family history of this disease [9]. Similarly, in Taiwan, through serological testing for EBV markers, it has been proven that individuals with a family history of nasopharyngeal carcinoma are more susceptible to the disease than the general population [10,11,12]. In research on the association between genetic factors and NPC, the primary focus is on investigating the genetic polymorphism of the human leukocyte antigen (HLA), which is necessary for presenting foreign antigens to immune lysis. In China and Asia, people with HLA-A2, B46, and B17 have 2–3 times the NPC risk compared to other HLA types [13]. Whereas in Caucasians, HLA-B5 is found to be associated with a lower risk of NPC [14]. In another study, the presence of HLA-A11, B13, and B22 alleles is linked to a reduced risk of developing nasopharyngeal carcinoma, while individuals with HLA-A2, C11, B14, B17, and B46 alleles have been found to have a higher susceptibility to this disease [15,16].

For environmental factors, diet, smoking and drinking, and environmental exposures are the three most focused study topics. In South China, salt-preserved food, including salted fish, has been considered as a possible etiological factor for NPC. The Chinese population in China, and Chinese migrants in other countries, display a high incidence of NPC, suggesting the dietary consumption of certain ethnic groups might be a contributory factor in NPC progression [16]. To further analyze geographical factors, genetic factors are the main factors for NPC etiology in the low-incidence populations, while specific diet combined with internal genetic factors play a major role in NPC high-incidence regions [1,17,18,19,20]. In searching for the oncogenic mechanism behind this kind of diet, results suggested that the nitrosamines in salted food are the main culprit for NPC development, which was supported by the animal evidence that feeding rats with Cantonese salted fishes developed carcinoma on the nasal cavities [21,22,23,24]. Nitrosamine is identified as a potent carcinogen [25,26]. Continuous consumption of such foods helps the accumulation of nitrosamine in the nasopharynx region. Moreover, some nitrate-reducing bacteria grow and metabolize the nitrates and nitrites of salty foods to N-nitroso compounds (NOCs) including N-nitrosopyrrolidine (NYPR), N-nitrosodimethylamine (NDMA), N-nitrosodiethylamine (NDEA), and N-nitrosopiperidine. These volatile NOCs are classified as probably carcinogenic to humans (group 2A) [27] and are also identified to be present in foods from high-incidence areas of NPC [28,29]. NOCs were implicated in the carcinogenesis of NPC through their genotoxicity and activation of oncogenic pathways [30,31,32]. Moreover, N-methyl-N’-nitro-N-nitrosoguanidine (MNNG, a kind of NOC) has been found to activate the EBV in NPC cell lines by itself, or cooperating with chemicals, and then increase subsequent NPC progression [33,34]. 

When it comes to the impact of viral factors on the development of nasopharyngeal carcinoma, EBV and HPV are two of the most studied viruses. The association of EBV with the origins of nasopharyngeal carcinoma has been extensively studied and confirmed in recent years, while the association with HPV is still under research. After the first demonstration of the association between EBV and NPC by Wolf et al. [35], abundant seroepidemiological studies revealed that elevated titers of antibodies against EBV latent and lytic antigens are detected in NPC patients, which are developed as diagnostic markers of NPC [36,37,38,39,40]. The residents in NPC high-risk areas have higher titers and frequencies of antibodies against EBV lytic antigens [10]. The retrospective study also revealed that the antibody occurred 2 to 28 months prior to the diagnosis of the disease [41]. Another meta-analysis suggested that the relative risks of developing NPC in individuals positive for antibodies against EBV are 85.3 and 32.7 times higher than those that are negative at 5 and 16 years, respectively [39]. On the other hand, EBV DNA was detected in NPC tissues, which were also developed as a precision marker of NPC detection, and various EBV lytic gene products were expressed. These numerous studies all support the etiological role of EBV in the development of NPC.

More recently, the novel etiological hypothesis of “NPC ecology theory” has been proposed [42]. Luo suggested that NPC is not a purely genetic disease but rather a disease that integrates genetic evolution and cancer ecological concepts. Various factors related to NPC play roles in environmental setup and evolutionary driving forces within this NPC ecosystem to achieve the effect of survival of the fittest, eventually driving the progression of NPC (Luo, 2023). This novel concept provides a comprehensive framework for understanding the causes of NPC, offering a new direction for understanding its development and cancer treatment.

## 2. The Treatment of NPC

Based on the staging system of the American Joint Committee on Cancer, NPC is clinically categorized into the following stages: 0, I, IIa, IIb, III, IVa, IVb, and IVc [43]. Appropriate treatments are applied for different stages. 

The standard treatment of NPC is a combination of radiotherapy and chemotherapy. For stages 0, I, and IIa, irradiation treatment is the primary choice, while chemotherapy is the preferred treatment at stages IIb, III, and IV. The most commonly used chemotherapeutics are cisplatin and 5-fluorouracil.

### 2.1. Radiotherapy

Radiation therapy (RT) is a traditional treatment of NPC because of its radiosensitivity and limited anatomic location. In recent years, there has been an advancement from traditional two-dimensional radiotherapy (2DRT) and three-dimensional radiotherapy (3DRT) to intensity-modulated radiotherapy (IMRT). Compared to conventional RT, IMRT can better cover the target area of the tumor and protect adjacent organs. In clinical surveys, IMRT showed an improved efficacy of treatment with better overall survival (OS), less toxicity, and reduced side effects compared with 2DRT or other conventional RTs [44,45,46]. In addition, the practicality of intensity-modulated proton therapy (IMPT) in NPC treatment has also been evaluated. Clinically, IMPT has better potential than IMRT in its tumor coverage and the reduction of the integral dose to organs at risk (OARs) and non-specific normal tissues [47]. Moreover, in comparison with helical tomotherapy (HT) treatment, apart from excellent tumor coverage and homogeneity, IMPT has better protection against OARs, especially at medium-to-low dose levels [48].

### 2.2. Chemotherapy

Chemotherapy is generally applicable for treating NPC beyond stage I due to its high versatility. It can be used alone or in combination with other types of treatments. For example, when combined with radiation therapy, the resulting chemoradiotherapy (CRT) approach is currently the standard treatment for mid to late-stage NPC. 

Until now, concurrent CRT has been the standard therapeutic approach for patients with stage III–IV non-metastatic NPC, which was reported first by the Al-Sarraf group [49]. In this phase III trial conducted in a non-endemic region, it was demonstrated that the combination of concurrent and adjuvant chemotherapy with radiotherapy can bring excellent OS benefits. Subsequent clinical trials have also confirmed the remarkable efficacy of this combination therapy [50,51,52,53,54]. 

Similarly, in other phase III studies, concurrent CRT alone also obtained better OS than that of radiotherapy alone [55,56]. In the meta-analysis conducted by the MAC-NPC group, concurrent CRT with/without adjuvant chemotherapy obtained a superior OS over radiotherapy alone [57]. For the drug usage of concurrent CRT, cisplatin is the most commonly used drug in most trials, while carboplatin [58,59], nedaplatin [60], and oxaliplatin [61] can also provide a decent efficacy but are less frequently used. 

For patients with a high-risk grade of recurrence NPC (≥T3N0), concurrent chemoradiation with either induction chemotherapy (ICT) or adjuvant chemotherapy (ACT) is recommended in the multidisciplinary treatment program. In the NCCN guidelines, induction chemotherapy (ICT) or adjuvant chemotherapy (ACT) are recommended for use in high-risk grades of NPC (≥N1 or ≥T3). For the patients requiring ICT treatment, three cycles of gemcitabine plus cisplatin are recommended because this regimen is easier to handle than others, has an improved OS, and has controllable toxicity [62,63]. Other alternative ICTs are also applied, including cisplatin plus either docetaxel, fluorouracil, or capecitabine. On the other hand, for patients with advanced NPC, concurrent CRT plus adjuvant chemotherapy is also recommended. This approach increased OS, and when compared with concurrent chemoradiation plus adjuvant chemotherapy, it showed a better OS than radiation therapy alone [57,64].

### 2.3. Immunotherapy

Nasopharyngeal carcinoma is a type of cancer that is highly infiltrated with lymphocytes and filled with stroma. It is a cancer highly associated with the Epstein-Barr Virus (EBV), and the virus also provides its own mechanisms for immune evasion to help cancer cells escape from immune surveillance. The EBV presents in a latency II state within nasopharyngeal carcinoma cells. In this state, only a limited number of viral proteins are expressed to reduce immunogenicity. Additionally, cancer and immune cells express a large number of immune checkpoint molecules, including PD-L1, PD-1, CTLA4, TIGIT, LAG3, and TIM3; the EBV also stimulates the expression of these molecules, thereby inhibiting the cytotoxic abilities of immune cells, including CD8+ and CD4+ T cells [65].

The immunotherapies for NPC patients are categorized into two types: EBV-targeting and non-EBV-targeting approaches. EBV-targeting immunotherapy includes the adaptive transfer of autologous and allogenic cytotoxic T-lymphocytes (CTLs) against EBV-associated antigens or dendritic cells-based therapy [66]. Non-EBV-targeting immunotherapies include antibody-based therapy (monoclonal or bispecific antibody), antibody-drug conjugated approaches, and engineered immune cell-based therapy (gene-knockout T-cells, chimeric antigen receptor T-cell therapies, or gene-knockout T-cells). The objective tumor responses in most of these immunotherapies occur only in a few cases of patients with advanced NPC, and they are still in the research or clinical trial phase. Among them, the most notable is the immune checkpoint antibody therapy, especially anti-PD-1 or anti-PD-L1 antibodies [67]. 

Up to now, several PD-1 and PD-L1 antibodies and bispecific antibodies for the treatment of nasopharyngeal carcinoma are undergoing clinical evaluation [68,69,70,71,72]. In addition, combination therapies of immune checkpoint therapy with other treatments, e.g., chemotherapy, seem to be more effective [73,74]. These results indicate that the application of an immune checkpoint inhibitor (ICI) in the treatment of NPC requires a more comprehensive assessment.

### 2.4. Therapeutic Vaccines

Because NPC cells contain foreign EBV antigens, EBV proteins are, therefore, a good target for vaccine development. There are two approaches for NPC vaccines. One is by injecting dendritic cells (DCs) processed with an LMP2 peptide into the inguinal lymph nodes of NPC patients, which induces specific CD8+ T cell activity and leads to a significant reduction in the tumor [75,76]. Another approach is to use recombinant vaccinia virus expressing the fusion protein of EBNA1/LMP2 to activate specific T-cell responses for killing cancer cells. This method has been found to be an effective and safe anticancer therapy in phase II clinical trials [77,78]. In general, NPC vaccines designed against EBV peptides have advantages such as safety and good efficacy. Attempts to use other EBV peptides as targets, such as gp350, LMP1/2, or gH/gL, are currently under development for NPC vaccine therapy.

### 2.5. Future Treatment

The standard treatment for early-stage nasopharyngeal carcinoma (NPC) is chemotherapy combined with radiotherapy, which generally has good outcomes. However, for advanced or recurrent nasopharyngeal carcinoma cases, the effectiveness is not very good. So far, there is still no effective treatment for such patients, suggesting developing various new therapies is very important. In addition to the three main therapies mentioned above, some emerging therapies under research also show considerable potential.

#### 2.5.1. miRNA-Based Therapy

miRNA-based therapy is a future treatment attracting a lot of attention. Its advantage is that it can target multiple pathways simultaneously and is easily combined with other treatments for concurrent use. In NPC, miRNAs are dysregulated to change their specific targets and involve tumorigenesis, metastasis, angiogenesis, and resistance to radio- and chemotherapies. Particularly, the miRNAs encoded by the EBV also heavily affect the tumorigenic properties of NPC. These characteristics enable miRNA not only to be used as a target for treatment but also as a diagnostic tool or an indicator for assessing the effectiveness of therapy. For example, miR-663 and miR-125b were shown to induce NPC cell proliferation, while miR-135a and miR-25 suppressed cell proliferation of NPC. Moreover, miR-29c and EBV-miR-BART6-3p could suppress the metastasis of NPC cells by targeting TIAM1 and lncRNA LOC553103 [79,80]. Up to now, a large number of miRNAs related to the carcinogenic process of nasopharyngeal carcinoma have been identified. The next challenge is to solve the delivery system of miRNAs, as well as the toxicity and off-target problems due to their broad targeting.

#### 2.5.2. Nanoparticle (NP)-Based Technology

Nanomedicine is currently receiving more attention for the application of cancer diagnosis and therapy. Nanoparticle (NP)-based technology is currently a very commonly used tool in nanomedicine. NPs can follow various regimens to deliver loaded drugs into cancer cells, allowing more drugs to enter the target cells and kill them more efficiently. Targeted NPs have guided properties, enabling precise drug delivery to the target cells, thus minimizing the impact on normal cells and achieving the goal of precision medicine. The nanoparticles loaded with folic acid, gefitinib and yttrium 90, or curcumin exhibited significant cytotoxicity against NPC cells with low toxicity to body weight of mice, suggesting this may be a potential treatment for NPC therapy [81,82]. 

#### 2.5.3. Exosome-Based Cancer Therapy

Exosome-based cancer therapy, another tool of nanomedicine, is a currently emerging treatment. Exosomes are a kind of extracellular vehicle (EVs) with 30–140 nm secreted by various kinds of cells. Until now, exosomes have been applied as a biomarker for diagnosis, exosome therapy, and drug delivery vesicles. Exosome-based therapies were shown to have additional advantages in combination with other treatments. The γδ T-secreting exosomes synergistically enhanced the efficacy of tumor killing against radioresistant NPC cells combined with radiotherapy [83].

## 3. Natural Compounds and Emodin

Natural compounds have always been one of the popular methods for treating diseases from ancient times to the present because of their safety and convenience. In recent years, they have been extensively used in cancer prevention and treatment. In cancer therapy, in addition to directly killing cancer cells, natural compounds are often used in adjunctive or preventive treatments. Their multi-targeted and less side-effect characteristics make them effective in treating chemoresistant tumors. 

### 3.1. Curcumin

Curcumin is the principal curcuminoid of turmeric, a member of the ginger family, Zingiberaceae. Curcumin is well known for its potent anti-inflammatory and antioxidant activities as well as anti-tumor and anti-metastasis properties. Curcumin exhibited an inhibitory effect on NPC proliferation through the induction of apoptosis [84], disruption of oncogenic signaling [85], dysregulation of miRNA [86,87], activation of autophagy [88], and even inhibition of EBV activity [89]. 

### 3.2. Epigallocatechin-3-Gallate (EGCG)

EGCG is another famous natural compound with antioxidant and anti-tumor activities. EGCG displayed a significant anti-tumorigenic activity on NPC tumorigenesis through the inhibition of EMT property [90], suppression of the transcriptional activity [91], interference with the regulation of miRNAs [92,93,94], and the inhibition of anti-apoptosis activity [95].

### 3.3. Flavonoids

Flavonoids are a large group of polyphenol compounds and have been proven to have potential anti-cancer, anti-inflammatory, antioxidant, and anti-virus activity in various diseases. For their anti-NPC activity, quercetin displays anti-proliferative activity in NPC cells by inducing cell cycle arrest and causing a synergistic effect with cisplatin [96]. Trifolirhizin inhibits cell proliferation, migration, and invasion in NPC cells through inhibition of PI3K/Akt signaling [97]. Luteolin was identified to suppress tumorigenic characteristics by disrupting oncogenic pathways, blocking the cell cycle, and inhibiting EBV reactivation harboring in NPC cells [98,99,100,101] In addition, emodin, another type of natural compound, shows remarkable anti-cancer capabilities, including NPC, and has attracted considerable attention in recent years. We will discuss this in detail in the following section. Up to now, the anti-cancer potential of natural compounds is still being continuously explored. 

### 3.4. Emodin

**Figure 1:**
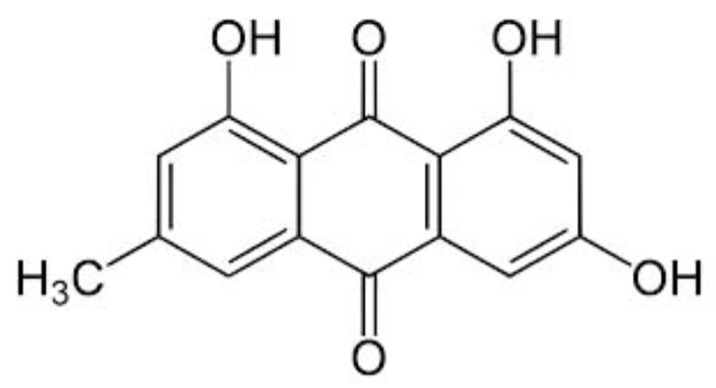
The chemical structure of emodin.

Emodin is an anthraquinone derivative, a type of naturally occurring phenolic compound (Figure 1). It is characterized by its orange color and is often found in the form of crystals. It is primarily derived from the roots and rhizomes of various plants and fungi, such as *Rheum palmatum* (a Chinese rhubarb), *Polygonum cuspidatum* (a Japanese knotweed), and some species of Aloe. Traditional Chinese medicine has utilized these plant sources for their medicinal properties for centuries.

Emodin exhibits a wide range of biological activities. These include anti-inflammatory, antimicrobial, and liver-protective effects. However, its potential anti-cancer effects have garnered significant scientific interest.

Emodin has been studied for its ability to inhibit the growth and spread of various types of cancer cells. It is believed to induce apoptosis in cancer cells, inhibit cell proliferation, and interfere with several signaling pathways involved in cancer development. Emodin also shows potential in sensitizing cancer cells to chemotherapy and in overcoming drug resistance.

#### 3.4.1. The Potential Mechanisms for Anticancer Properties of Emodin

**Figure 2:**
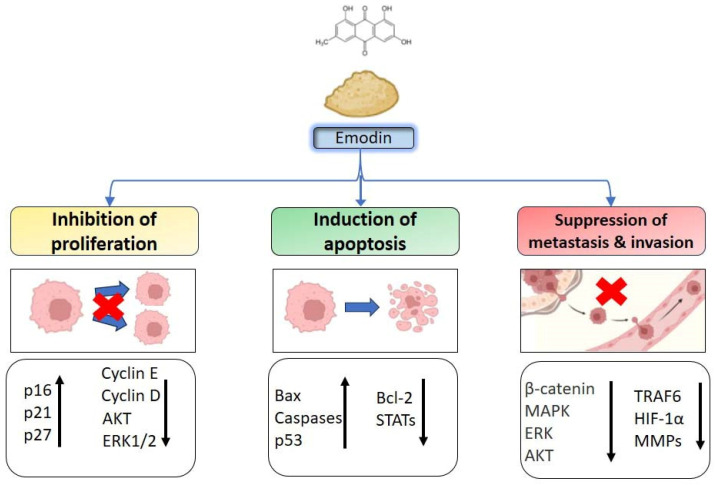
The potential mechanisms for anticancer properties of emodin.

Emodin, a naturally occurring compound found in certain plants, exhibits notable anti-tumor activity through various mechanisms. Understanding these mechanisms is crucial for exploring their potential in cancer therapy. The anti-tumor mechanisms of emodin are described as follows (Figure 2).

##### Inhibition of Cancer Cell Proliferation and Tumor Growth

Emodin has been found to inhibit cancer cell proliferation in vitro and tumor growth in vivo. Wang et al. found that various amounts of emodin treatment inhibited cell proliferation by the down-regulation of cell cycle regulators, Cyclin D and E, in cervical cancer-derived (Hela), choriocarcinoma-derived (JAR) and ovarian cancer-derived (HO-8910) cells. Emodin also induced apoptosis and autophagy and inhibited angiogenesis in these cells [102]. Several studies also found that emodin-induced cell cycle G2/M arrest restrains cell proliferation and disrupts mitotic activity [103,104].

In addition to the disruption of the cell cycle, emodin regulated the function of tumor suppressor genes and inhibited cell growth. The bioinformatic and experimental analysis identified that emodin affected p53 expression and phosphorylation of PI3K/AKT to inhibit cell growth in a diffuse large B-cell lymphoma [105].

Emodin treatment inhibited cell growth by inhibiting VEGFR2-AKT-ERK1/2 pathways and increasing miR-34a expression, a key regulator of p53 networks in a HepG2 cell model [106] and colon cancer cells in a HCT-116 model [107]. 

##### Induction of Apoptosis

Apoptosis, known as programmed cell death, is a critical biological process in which cells undergo an orderly and controlled sequence of events leading to their self-destruction. Inducing apoptosis is also an important means of eliminating cancer. Large numbers of studies have revealed that emodin can induce apoptosis (programmed cell death) in cancer cells. It modulates the expression and activity of key proteins involved in the apoptotic pathways, such as Bcl-2, Bax, caspases, and p53. This leads to the activation of the intrinsic and extrinsic pathways of apoptosis, effectively causing cancer cells to self-kill. 

Emodin was found to activate TRIB3/NF-κB signaling and induce ER stress-mediated apoptosis in human non-small cell lung cancer (NSCLC) cell lines (A549 and H1299) [108]. In the HCC cell lines SMMC-7721 and HepG2, emodin causes apoptosis by suppressing various signal pathways, such as PI3K/AKT signaling [109] and ERK phosphorylation [110]. In addition to interfering with apoptotic signal pathways, emodin also dysregulates the expression of BCL-2 family genes and leads to cell apoptosis [111].

Autophagy is a fundamental cellular process where cells degrade and recycle their own components. It has a complex role in cancer development with both pro-survival and pro-apoptotic activities, depending on the cellular conditions. Emodin has been found to induce autophagy in the colon cancer cell line CoCa [111] and in the cervical cancer cell line HeLa [112] by different pathways. 

##### Suppression of Tumor Metastasis and Invasion

Tumor metastasis and invasion are hallmarks of cancer development, which is correlated with poor outcomes for cancer patients, including poor prognosis, poor survival rates, drug resistance, and tumor relapse [113]. Emodin was found to reverse TGF-β-induced EMT and resulted in the suppression of metastasis and invasion through miRNA regulation [114,115,116]. In addition, emodin treatment was reported to inhibit cell growth, EMTn, and cell invasion by suppressing Wnt/β-catenin signaling in ovarian cancer and colon cancer cell lines [117,118]. 

Besides these, the TNF receptor-associated factor 6 (TRAF6) is well-known to be a crucial factor in matrix metalloproteinase (MMP) expression, facilitating cell metastasis, and in VEGF expression contributing to angiogenesis. Adequate emodin treatment inhibited both TRAF6/HIF-1α/VEGF and TRAF6/CD147/MMP9 axes to block cell metastasis and angiogenesis in human anaplastic thyroid cancer cell lines in vitro and in vivo [119]. Moreover, by suppressing other ERK/MAPK and PI3K/AKT signal pathways, emodin treatment inhibited cell metastasis and invasion in HCC MHCC-97H cell lines [120]. 

#### 3.4.2. Research Progress of Emodin Treatment on NPC Therapy

A large number of comprehensive studies have demonstrated that emodin has great potential in cancer therapy, which is well documented in different review articles. However, as an important cancer type in Southeast Asia, there are few articles that compiled corresponding studies on the use of emodin in the treatment of NPC.

For the treatment of NPC, Ma et al. demonstrated that emodin treatment inhibited more significant cell proliferation and induced more profound apoptosis and cell cycle arrest in poorly differentiated human nasopharyngeal carcinoma cells (CNE-2Z) than normal epithelial nasopharyngeal NP69-SV40T cells [121]. Moreover, they found that emodin activates chloride currents to induce cell apoptosis in CNE-2Z cells but not in NP69-SV40T cells. These activated chloride currents can also be inhibited by chloride channel blockers and prevent cells from undergoing apoptosis, indicating that the chloride channel may be the potential target molecular of emodin exerting its anti-tumor efficiency in NPC therapy [122]. 

In another study, Hou et al. reported that emodin treatment could enhance the radiosensitivity of NPC CNE-1 cells in vitro and in vivo models. Combination treatment with radiation and emodin significantly increased cell apoptosis, G2/M cell cycle arrest, and reactive oxygen species (ROS) production, while it decreased the expression of HIF-1α compared to that of radiation or emodin treatment alone (Hou et al., 2013) [122]. 

NPC has one unique characteristic that differs from other cancers in that cancer cells harbor the EBV. This harboring of the EBV may provide advantages in oncogenic force and immune evasion, which may lead to the EBV as an alternative target in NPC therapy. 

In our laboratory, we have demonstrated that EBV reactivation induced profound genomic instability and tumorigenesis in vitro and in vivo [123,124]. Several lytic proteins of the EBV were identified to induce DNA mutation, chromosome aberrations, and genomic instability, which can contribute to NPC tumorigenesis [125,126,127]. If we blocked EBV reactivation through Zta siRNA or flavonoids, the tumorigenic properties and tumor growth of NPC were repressed in vitro and in vivo [101,123,127]. Similarly, we treated the EBV-positive cell lines, NA and HA cells, with various concentrations of emodin, the EBV reactivation was inhibited, and several oncogenic signatures were also reduced. Moreover, a decrease in tumor growth in mouse models has also been found, suggesting that emodin repressed NPC tumorigenesis by anti-EBV activity [128]. In addition, in our recent finding, emodin could docked into the active site of EBV DNase, an EBV lytic protein playing a major role in genomic instability, which caused the suppression of DNase-induced genomic instability [129] In the field of evaluation of emodin on NPC treatment, although so far there have not been many studies, they have shown the therapeutic potential of emodin, making it worthy of further research.

#### 3.4.3. The Treating Efficacy of Other Anthraquinone Derivatives on NPC Therapy

In addition to emodin, other anthraquinone derivatives were also reported with anti-NPC activity. Aloe-emodin is a specific type of emodin found mainly in aloe vera plants. It was found to inhibit NPC cell growth [130] and induce NPC cell apoptosis through the caspase-8-mediated death pathway [131]. It was also reported to inhibit the invasion of NPC cells through the p38 MAPK-NF-κB-dependent pathway [132]. In addition, Aloe-emodin targets multiple signaling pathways to suppress tumor growth in NPC cells [133]. Physcion, another compound of anthraquinone derivative, induces apoptosis and autophagy in NPC cells [134] Moreover, 1,8-dihydroxy-3-acetyl-6-methyl-9,10 anthraquinone exhibits potent radiosensitizing in NPC cells, suggesting its application in adjuvant radiotherapy [135,136].

## 4. Conclusions: The Potential and the Challenge of Emodin for Clinical Applications of NPC

**Figure 3:**
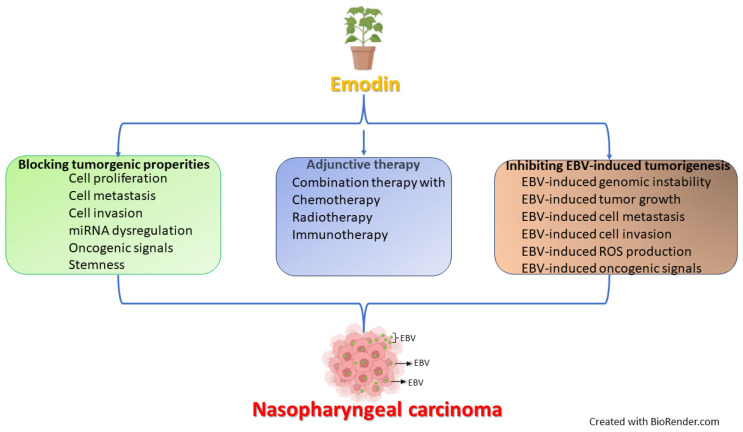
The possible mechanisms of emodin to fight against NPC tumorigenesis. Emodin possesses three capabilities to reduce NPC tumorigenesis. First, emodin can inhibit various oncogenic characteristics of NPC cells. Second, it can serve as an adjunct to other primary treatments for NPC. Third, it can reduce the tumorigenic effects induced by the EBV by inhibiting its activation.

In summary, emodin has demonstrated a variety of anti-cancer mechanisms in the treatment of other types of cancer. Although there were few studies exploring these mechanisms in nasopharyngeal carcinoma cells, it is reasonable to speculate that they would also occur in NPC cells. In addition, the treatment of NPC with emodin has several advantages (Figure 3). For example, the mechanisms of emodin’s regulation of chloride ion channels and its effectiveness against the EBV are rarely found in the treatment of other types of cancer. This not only adds more available weapons for combating NPC but also contributes to addressing treatment resistance in cancer. Moreover, because the primary treatment of NPC is the combination of radio and chemotherapy, the combination of emodin and radiotherapy causes synergistic effects in NPC treatment, suggesting emodin can serve as an alternative option for adjunctive therapy (Figure 3).

On the other hand, although current research indicates that emodin’s anti-cancer capabilities are promising, there are several challenges to overcome before it can be clinically applied. First, although emodin’s ability to inhibit cancer is unquestionable, many studies lack detailed mechanisms, and this area still requires more effort. Second, the biological toxicity of emodin needs to be addressed. Limited animal experiments on toxicity have shown that emodin has potential hepatotoxicity and genotoxicity, although these reports are somewhat contradictory. This aspect needs clarification. Additionally, the bioavailability of emodin is also a challenging problem to overcome. The chemical properties of emodin make it difficult for cells to absorb, which also affects its clinical application benefits. 

In the future perspective for the application of emodin, combination therapy is currently the most promising direction. Whether it is emodin combined with immunotherapy, chemotherapy, or even emerging therapies, it can reduce the side effects of emodin and amplify its cytotoxic benefits, forming a complementary approach with various treatments. This is also a new direction in all cancer therapies. Nevertheless, emodin remains a worthy target to continuously research and develop in the treatment of NPC.
